# Monitoring the skin structure during edema *in vivo* with spatially resolved diffuse reflectance spectroscopy

**DOI:** 10.1117/1.JBO.28.5.057002

**Published:** 2023-05-13

**Authors:** Denis A. Davydov, Gleb S. Budylin, Alexey V. Baev, Daniil V. Vaypan, Elena M. Seredenina, Simon T. Matskeplishvili, Stanislav A. Evlashin, Armais A. Kamalov, Evgeny A. Shirshin

**Affiliations:** a Lomonosov Moscow State University, Faculty of Physics, Moscow, Russia; b First Moscow State Medical University, Biomedical Science and Technology Park, Laboratory of Clinical Biophotonics, Moscow, Russia; c Moscow State University, Medical Research and Education Center, Moscow, Russia; d Institute of Spectroscopy of the Russian Academy of Sciences, Moscow, Russia; e Skolkovo Institute of Science and Technology, Center for Materials Technologies, Moscow, Russia

**Keywords:** spatially resolved diffuse reflectance spectroscopy, water in the skin, dermal thickness, hypodermal thickness, cutaneous edema

## Abstract

**Significance:**

Edema occurs in the course of various skin diseases. It manifests itself in changes in water concentrations in skin layers: dermis and hypodermis and their thicknesses. In medicine and cosmetology, objective tools are required to assess the skin’s physiological parameters. The dynamics of edema and the skin of healthy volunteers were studied using spatially resolved diffuse reflectance spectroscopy (DRS) in conjunction with ultrasound (US).

**Aim:**

In this work, we have developed a method based on DRS with a spatial resolution (SR DRS), allowing us to simultaneously assess water content in the dermis, dermal thickness, and hypodermal thickness.

**Approach:**

An experimental investigation of histamine included edema using SR DRS under the control of US was conducted. An approach for skin parameter determination was studied and confirmed using Monte-Carlo simulation of diffuse reflectance spectra for a three-layered system with the varying dermis and hypodermis parameters.

**Results:**

It was shown that an interfiber distance of 1 mm yields a minimal relative error of water content determination in the dermis equal to 9.3%. The lowest error of hypodermal thickness estimation was achieved with the interfiber distance of 10 mm. Dermal thickness for a group of volunteers (7 participants, 21 measurement sites) was determined using SR DRS technique with an 8.3% error using machine learning approaches, taking measurements at multiple interfiber distances into account. Hypodermis thickness was determined with root mean squared error of 0.56 mm for the same group.

**Conclusions:**

This study demonstrates that measurement of the skin diffuse reflectance response at multiple distances makes it possible to determine the main parameters of the skin and will serve as the basis for the development and verification of an approach that works in a wide range of skin structure parameters.

## 1 Introduction

The impaired balance of water distribution in the human organism accompanies numerous pathological states, and measuring water content in tissues is required for biomedical diagnostics. For instance, during heart failure,^1^ physical examination using the “pitting scale” and weighing the patient remains the most useful approaches for assessing edematous syndrome; however, these methods are subjective and insufficient.^2^ Assessment of water and lipids content in breast cancer tissues can provide important information about the characteristics of a tumor,^3^ as the high water content in a tumor indicates edema and increased cellularity.^4^^,^^5^ Likewise, the measurement of hydration in different skin layers is interesting for dermatology and cosmetology:^6^ for instance, water content in the dermis is a marker of changes in the elastic properties of the skin,^7^ and hydration of the stratum corneum is indicative of its barrier function.^8^ Hence, the necessity of water content assessment stimulates the development of appropriate techniques, including those based on optical spectroscopy.^9^

Optical methods based on molecular contrast, i.e., spectroscopic signatures of water molecules, have been numerously suggested in the literature as fast, sensitive, and non-invasive tools for assessing hydration *in vivo*. Confocal Raman and THz spectroscopy are mainly applied for studying the skin’s upper (epidermis) layer.^10^^,^^11^ Near infrared (NIR) spectroscopy and imaging allow water content assessment in deeper layers (dermis and hypodermis).^3^^,^^12^^,^^13^ For instance, in heart failure patients, peripheral edema was analyzed with short-wave NIR imaging.^2^ Cutaneous edema induced by histamine application was studied with diffuse reflectance spectroscopy (DRS),^9^^,^^14^ Raman fiber probe,^9^ and multispectral NIR imaging.^2^^,^^9^^,^^15^ The question of choosing the optimal distances between the source and the detector for leg edema evaluation were discussed in Nanjo et al.^14^

**Fig. 1:**
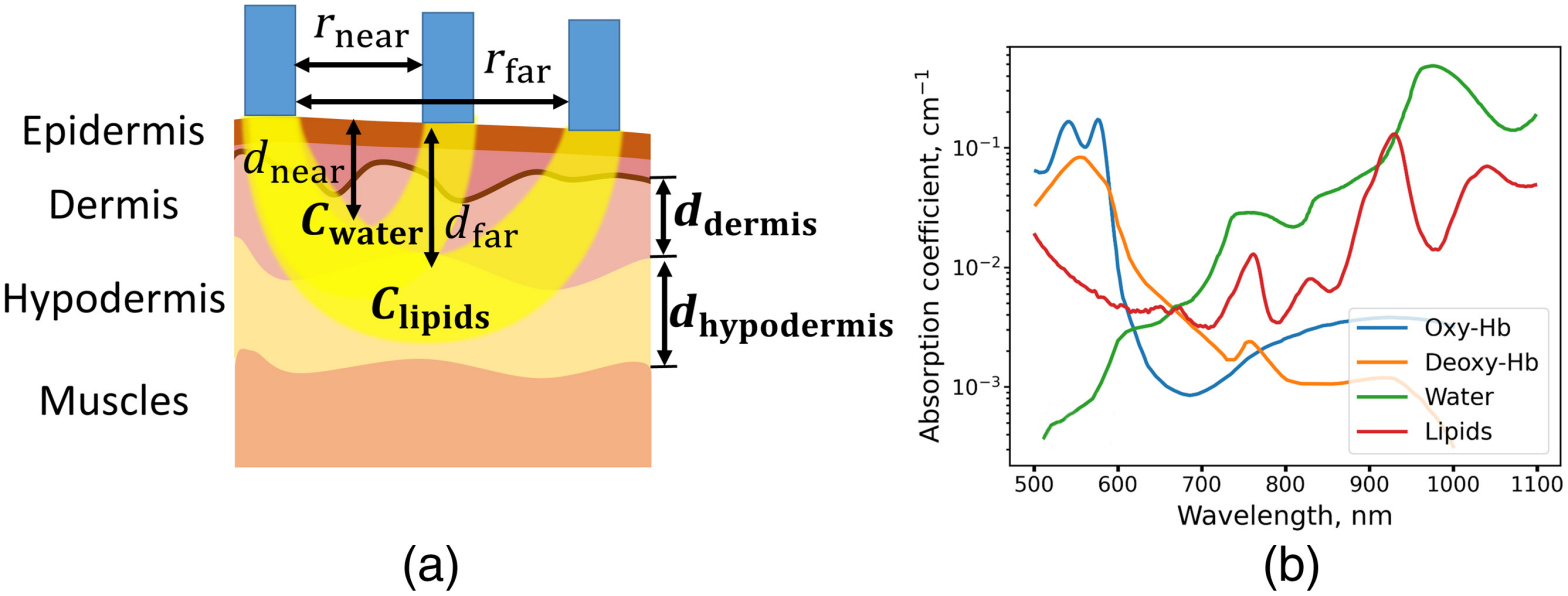
(a) Schematic representation of the SR DRS method for the skin measurements *in vivo* and main parameters determining the optical response: water concentration in the dermis $c_{\text{water}}$, dermal thickness $d_{\text{dermis}}$, lipids content in the hypodermis $c_{\text{lipids}}$, and hypodermal thickness $d_{\text{hypodermis}}$. (b) Absorption spectra of the main skin chromophores: hemoglobin (Deoxy-Hb), oxyhemoglobin (Oxy-Hb), water, and lipids.

Since the optical and morphological properties of the skin differ significantly,^16^ the assessment of hydration and cutaneous edema of the skin by optical methods is a complicated task. The top layer of the skin is the epidermis, 50 to 100  μm thick. Under the epidermis, there is the dermal layer, 1 to 2 mm thick. Dermis is mainly composed of collagen, which, along with elastic fibers, forms the extracellular matrix responsible for the viscoelastic properties of the skin.^17^ Dermis contains blood capillaries; increased capillary permeability, e.g., during inflammation or heart failure, may result in interstitial fluid retention.^18^ Below the dermis is the hypodermis, or the subcutaneous layer, which consists primarily of the fat tissue. There is a layer of muscle under the hypodermis. Its optical properties differ significantly from those of the hypodermis due to the higher water concentration and the absence of lipids in which the CH absorption band is more pronounced. The difference in the optical properties of the layers makes it possible to separate their contribution to the reflectance signal using spatially resolved DRS. Hence, when measuring the diffusely reflected NIR signal from the skin, water in the dermis and lipids in the hypodermis contribute to the spectrum. In this paper, we aim to simultaneously determine the molecular (water content in the dermis and lipid content in the hypodermis) and morphological (thickness of the dermis and hypodermis) properties of the skin (Fig. 1) *in vivo* using the spatially resolved DRS (SR DRS).

SR DRS is based on measuring spectra at varying source-detector distances, thus performing the depth scanning of a material.^19^ To solve the inverse problem, i.e., to determine the absorption parameters from SR DRS data, Monte Carlo modeling of light propagation in multi-layered materials followed by interpolation^20^^,^^21^ and machine learning^22^^–^^24^ can be used. This approach has already proven itself capable of determining the content of hemoglobin and melanin in tissues. However, the problem of determining water *in vivo* using this technique has not been considered previously. The SR DRS has also been applied for determining the thicknesses of tissue layers in *ex vivo* samples and optical phantoms^12^^,^^25^^,^^26^ thus making it a promising technique for sensing the properties of individual skin layers *in vivo*. This issue was also addressed in the present study.

Here, based on simulated and experimentally obtained SR DRS data, changes in water concentration and dermal and hypodermal layer thickness were investigated for the cases of cutaneous edema and normal skin. For calibration of parameters recovered from the DRS data, the thickness of the skin layers was measured by ultrasound (US) examination. The ability of the SR DRS to determine molecular and morphological parameters of the skin was evaluated experimentally. The presented study is an important step for the development of a fully optical technique that can be useful for fluid retention estimation, pathological skin conditions, and dehydration assessment. Examination of skin physiology in cosmetology and dermatology may benefit from this approach.

## 2 Materials and Methods

### 2.1 Diffuse Reflectance Spectra Measurement

**Fig. 2:**
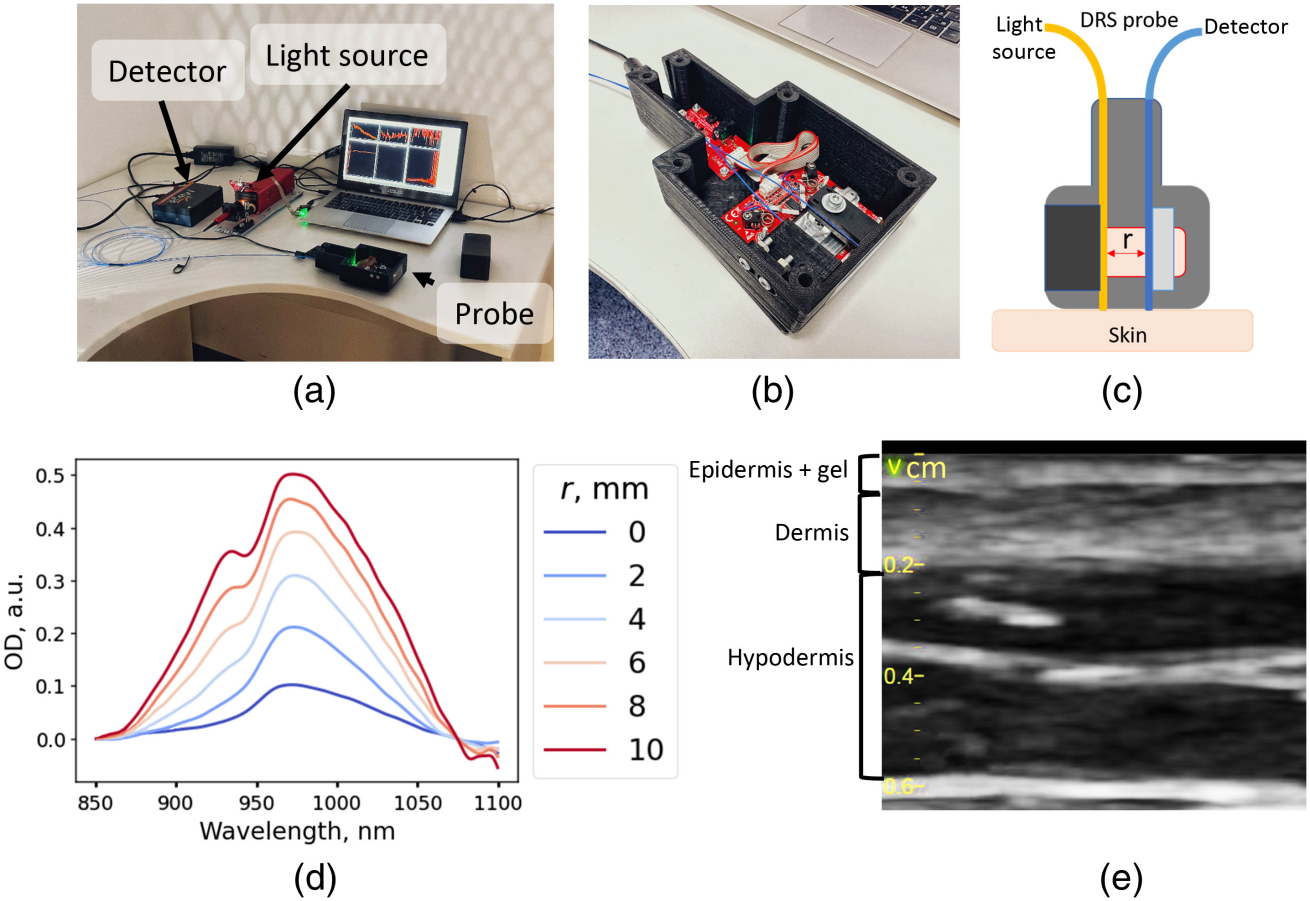
Images and schematic of the setup and a typical view of the experimental data. (a) Photo of the experimental setup, (b) photo of the SR DRS probe, (c) the scheme of the SR DRS probe, (d) representative diffuse reflectance spectra measured for distances between the source and the detector in the range from 0 to 10 mm, and (e) US images of the skin obtained with a linear 11L-D probe at a scanning frequency of 12 MHz using the Vivid E95 device.

The setup used in this work allowed SR DRS measurement in the 400- to 1100-nm wavelength range using optical fibers with a core diameter of 550  μm. The photo of the experimental setup is presented in Fig. 2(a), while the photo and scheme of the DRS probe are presented in Figs. 2(b) and 2(c). The distance between the fibers was varied using a linear translation stage with a resonant piezoelectric motor (ELL17/M Stage, Thorlabs, United States) in the range from 0 to 10 mm with a 0.5-mm step and positioning accuracy of 50  μm. Detection was performed with a Maya2000Pro spectrometer (Ocean Optics, United States), and the SLS201 (Thorlabs, United States) was used as the light source. The effective optical density (OD) spectrum was calculated as follows:

$$\mathrm{OD}(\lambda )=-\ln(\frac{I(\lambda )-I_{bg}(\lambda )}{I_{\mathrm{ref}}(\lambda )-I_{bg}(\lambda )}),$$ (1)

where I(λ) is the signal intensity of the sample, $I_{bg}(\lambda )$ is the background signal, and $I_{\mathrm{ref}}(\lambda )$ is the signal intensity from the reference sample (LabSphere, United States). Representative DRS spectra measured at different distances between the source and detector fibers are shown in Fig. 2(d). The optical fibers were located perpendicular to the skin surface in all experiments.

### 2.2 Processing of Diffuse Reflection Spectra

The OD spectra were fitted by a linear combination of the absorption spectra of the main components (water and lipids) and the baseline, which takes into account the contribution of the absorption of other chromophores (oxyhemoglobin, deoxyhemoglobin) and scattering:^9^^,^^27^^,^^28^

$$\mathrm{OD}(\lambda )=C_{0}+C_{1}\lambda +A_{\text{water}}\varepsilon_{\text{water}}(\lambda )+A_{\text{lipid}}\varepsilon_{\text{lipid}}(\lambda ),$$ (2)

where λ is the wavelength, $C_{0}$ and $C_{1}$ are related to contributions of background absorption and the scattering, $A_{\text{water}}$ and $A_{\text{lipid}}$ are the contributions of water and lipids absorption, $\varepsilon_{\text{water}}(\lambda )$ and $\varepsilon_{\text{lipid}}(\lambda )$ are the normalized absorption spectra of water and lipids. The coefficients were obtained by minimizing the difference between the fitted and experimental spectra using the least squares method.

### 2.3 Ultrasound Examination of the Skin

Variations in the thickness of the skin layers during edema were investigated using the Vivid E95 device (GE Healthcare, United States) with a linear 11L-D probe at a scanning frequency of 12 MHz. The skin images obtained using the US examination allowed the determination of the thickness of the dermis and hypodermis [Fig. 2(e)]. The measurements were taken for at least five vertical profiles for each image using the ImageJ software.^29^

### 2.4 Monte Carlo Simulation of Diffuse Reflectance Spectra

Light propagation in the skin was simulated using the GPU implementation of the Monte Carlo Method [Monte Carlo modeling of light transport (MCML)].^30^ The skin was modeled as a structure consisting of three layers with individual scattering and absorption properties. The first layer represented the dermis (where the water molecules are the main absorbers), the second layer simulated the hypodermis (with the lipids as the main absorber), and the third layer modeled the muscles (with the water as the main absorber). Using the simulation data, models were built to determine the water concentration in the dermis and the thickness of the dermis, as well as the thickness of the hypodermis. The determination of the parameters of the muscle layer was not carried out in the framework of this work.

The simulation was performed for varying values of the first $d_{\text{dermis}}$ and second $d_{\text{hypodermis}}$ layer thicknesses and absorption coefficients of all layers: $\mu_{a1}$, $\mu_{a2}$, and $\mu_{a3}$. The scattering coefficients of all layers were fixed to reduce the time required for simulations. The diffuse reflection coefficient R for each set of parameters was calculated as the ratio of the number of photon packets collected from the round-shaped region simulating an optical fiber with a diameter of 550  μm, exiting from the medium at all angles (solid angle of 2π steradian), to the number of photon packets entering the medium. For comparison, calculations were performed taking into account the finite numerical aperture of the fiber (data not shown), the dependences that were obtained for the simulated data did not change qualitatively [see Figures S1(a) and S1(b) in the Supplementary Material]. Effective OD for the simulated data was calculated as OD=−ln(R).^19^ The distance between the detector and light source varied in the range from 0.025 to 15 mm with a step of 0.05 mm. Thus, simulation distance range was wider than the range in experiment. For each layer, the value of the absorption coefficient $\mu_{\mathrm{ai}}$ (i=1,2,3) varied in the range from 0.001 to $10\;{\mathrm{cm}}^{-1}$ on a logarithmic grid with 20 nodes. Scattering spectra for all layers were assumed to be independent of wavelength in the considered simulation range. The scattering coefficient of the dermis was $200\;{\mathrm{cm}}^{-1}$, $100\;{\mathrm{cm}}^{-1}$ for hypodermis, and $90\;{\mathrm{cm}}^{-1}$ for muscles. The anisotropy factor g for each layer was equal to 0.9. The selected values correspond to typical values of the absorption and scattering coefficients of the skin.^31^ The thickness of the first layer, $d_{\text{dermis}}$, varied from 1 to 2.5 mm with a step of 0.5 mm. The values of thickness of the second layer $d_{\text{hypodermis}}$ were 0.5, 1, 2, 3, 4, and 5 mm. The third layer (muscle) thickness was equal to 5 mm. As a result, 240,000 MCML simulations were performed with $10^{7}\text{ photon}$ packets each.

For intermediate values of the absorption and scattering coefficients, the values were interpolated between the grid nodes by the K-nearest neighbors method. The following normalization was carried out to select the neighboring absorption and scattering coefficients:

$$\mu_{a}^{\log}=\ln(\mu_{a}),$$ (3)

$$\mu_{a}^{\text{norm}}=(\mu_{a}^{\log}-\min(\mu_{a}^{\log}))/(\max(\mu_{a}^{\log})-\min(\mu_{a}^{\log})).$$ (4)

Hence, each normalized value of the absorption and scattering coefficient was uniformly distributed and varied from 0 to 1. As the input data for the interpolation model, the absorption coefficients $\mu_{a1}^{\text{norm}}$, $\;\mu_{a2}^{\text{norm}}$, and $\mu_{a3}^{\text{norm}}$, the dermal thickness $d_{\text{dermis}}$, and the hypodermal thickness $d_{\text{hypodermis}}$ were used. The target variable of the model was the reflectance R(r) at a fixed distance. Based on the input parameters, the dataset was split into the training (95%) and test (5%) subsets. The optimal values for the number of neighbors were estimated using cross-validation on the training dataset.

The described approach made it possible to calculate the diffuse reflection spectra at different distances between the source and the detector for any combination of layers’ thicknesses and absorbers concentrations using the spectral dependencies of their absorption and scattering coefficients. Next, the algorithms for determining water concentration, as well as the dermal thickness and hypodermal thickness, were developed.

### 2.5 Simulation Configurations and Fitting

The dataset containing simulated DRS spectra of three-layered structures was obtained using the described algorithm (Sec. 2.4). The volume fraction of water W in the upper layer varied in the range from 0.5 to 1, the thickness of the upper layer (dermis) $d_{\text{dermis}}$ varied in the range from 1 to 2.5 mm, the thickness of the second layer (hypodermis) $d_{\text{hypodermis}}$ varied in the range from 0.5 to 5 mm and the volume fat fraction F in the second layer (hypodermis) was fixed to 1. The volume of water fraction $W_{3}$ in the third layer (muscles) was fixed to 0.8.

Simulated DRS spectra for each distance r between the fibers were fitted by Eq. (2), and the dependences of the fitting parameters $C_{0}$, $C_{1}$, $A_{\text{water}}$, and $A_{\text{lipid}}$ as a function of distance were obtained. Then, linear regressions for the fitting parameters against the input parameters were performed, and deviations of the predicted values from the true values were calculated. This approach allowed us to estimate the error of skin parameters determination obtained during the experiment. Two options were considered: (1) linear regression for the amplitudes at a single distance and (2) linear regression for all amplitudes at all source–detector separations.

The average relative error was calculated to assess the error of determining the input parameters from the linear regression for a single distance. The following calculations were carried out for the water volume fraction. For a distance r, the predicted water contribution $W_{\mathrm{pred}}$ was determined from the linear fit for $A_{\text{water}}$, and water content error $\delta_{W}(r)$ was calculated as

$$\delta_{W}(r)=\frac{1}{N}\overset{N-1}{\underset{i=0}{\sum }}\frac{\left|W_{pred}^{\left(i\right)}(r)-W^{\left(i\right)}\right|}{W^{\left(i\right)}},$$ (5)

where the index i denotes various input parameter configurations (W, $d_{\text{dermis}}$, and $d_{\text{hypodermis}}$) and N is the number of these configurations. Similar calculations were made for the predicted values of dermal thickness $d_{\text{dermis}}$ and hypodermal thickness $d_{\text{hypodermis}}$.

Dermal thickness and hypodermal thickness predictions were evaluated using both linear regression for $A_{\text{water}}$ and $\;A_{\text{lipid}}$ for a single distance and using general linear regression for $A_{\text{water}}$ and $A_{\text{lipid}}$ at all distances. Since these features are highly correlated and the machine learning procedure can lead to overfitting, the L2 regularization was carried out. After that, the model was trained on the spectra for a randomly sampled subset of input parameters, and the prediction error was evaluated on a test set that did not intersect with the training set.

### 2.6 Experimental SR DRS Data Fitting

The following approach was used for the interpretation of experimental diffuse reflectance spectra. US imaging allowed us to determine the thicknesses of the dermal and hypodermal layers while concentrations of molecular components in each layer remained unknown. Thus, linear regression between the amplitudes in Eq. (2) and the observed thicknesses was performed to analyze the possibility of experimental determination of the thickness from DRS data.

In the case of histamine-induced edema, when the dermal thickness was expected to vary, linear regression between $A_{\text{water}}$ and US-determined dermal thickness was analyzed. In the second experiment, the normal skin of seven volunteers (two males, five females) was studied. The mean age of volunteers was 23 years, the minimum and maximum ages were 21 and 27, respectively. A model was built based on the linear regression with L2 regularization and leave-one-out validation. The $A_{\text{water}}$ and $A_{\text{lipids}}$ amplitudes obtained for all source-detector distances were used as predictive features.

## 3 Results and Discussion

### 3.1 Assessment of the Cutaneous Edema in SR DRS

The SR DRS technique was applied to assess variations in the skin parameters caused by edema induced by histamine application. Similar models for edema were used in previous works.^10^^,^^17^

**Fig. 3:**
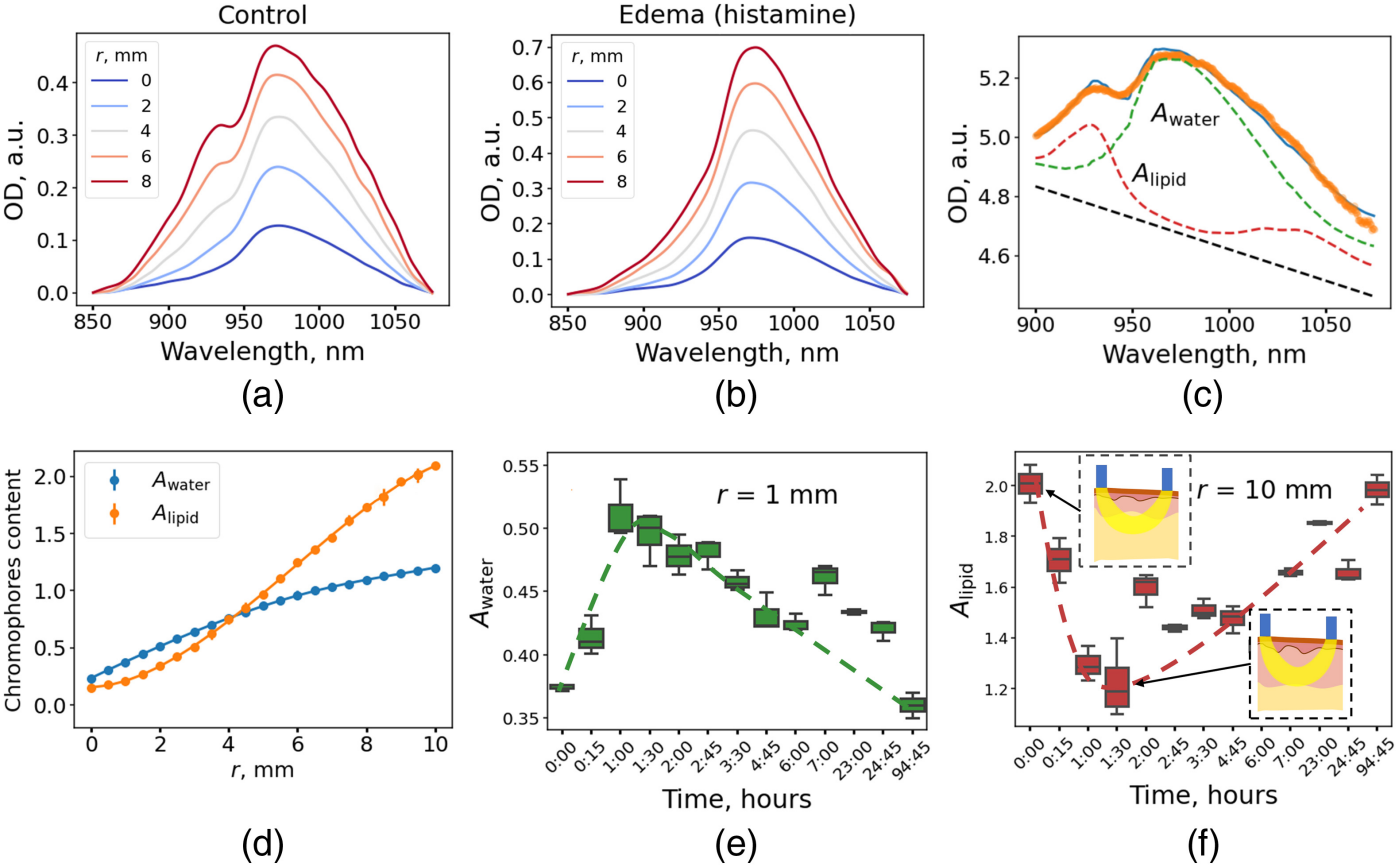
Experimental OD spectra for normal skin (a) and following histamine application (b) measured in the same skin area. The parameter r denotes the source-detector separation in panels (a) and (b). (c) Example of fitting the measured DRS data with Eq. (2). Orange line corresponds to experimental data, blue line corresponds to fit, and green and red dashed lines corresponds to water and lipid contributions to the spectrum, respectively. (d) Representative dependence of the water and lipids amplitudes ($A_{\text{water}}$ and $A_{\text{lipid}}$) on the distance between the source and the detector fibers. (e, f) Kinetics of the $A_{\text{water}}$ (e) and $A_{\text{lipid}}$ (f) amplitudes obtained for the r=1  mm and r=10  mm source-detector separation observed in the process of edema.

Typical DRS spectra of the skin exhibit the bands centered at 930 nm (mainly originating from lipids in the hypodermis) and at 970 nm (water) [Fig. 3(a)]. Histamine application to the skin led to a pronounced increase of the 970-nm band, accompanied by a decrease of the 930-nm band [Fig. 3(b)].

The OD spectra were fitted in the 900- to 1075-nm range to characterize the contribution of lipids and water [Eq. (2), Fig. 3(c)]. Thus, amplitudes $A_{\text{water}}$ and $A_{\text{lipid}}$ as functions of distance between source and detection fibers were obtained [Fig. 3(d)]. To characterize the change in the water content of the dermis, the spectra measured at small distances between the fibers were analyzed. This configuration corresponds to shallow detection depth, which reduces the contribution of the hypodermis. The maximum increase of $A_{\text{water}}$ during edema was 33±3% relative to the control. However, based on the confocal Raman spectroscopy data,^32^ the water content in the dermis can be estimated as ∼70%. Hence, the 33% increase means water content rises to as much as ∼90%. This value seems inadequate from the physiological point of view and, as shown below (Sec. 3.3), originates from the increase in dermal thickness.

The kinetics of the lipids absorption band $A_{\text{lipid}}$ was estimated for the DRS measured at a distance of 10 mm when the detection depth is large and light reaches the hypodermis. A decrease in the $A_{\text{lipid}}$ after edema induction was observed [Fig. 3(f)], which can be explained by the skin swelling and a concomitant decrease of the hypodermis contribution to the detection volume. The Monte-Carlo simulation of light propagation was performed to validate this hypothesis.

### 3.2 SR DRS Modeling for Different Structures of the Skin

Using the simulated SR DRS spectra of the three-layered skin model with varying parameters, we tested several approaches for the inverse problem solution, i.e., determination of (1) water content in the dermis, (2) dermal, and (3) hypodermal thickness.

**Fig. 4:**
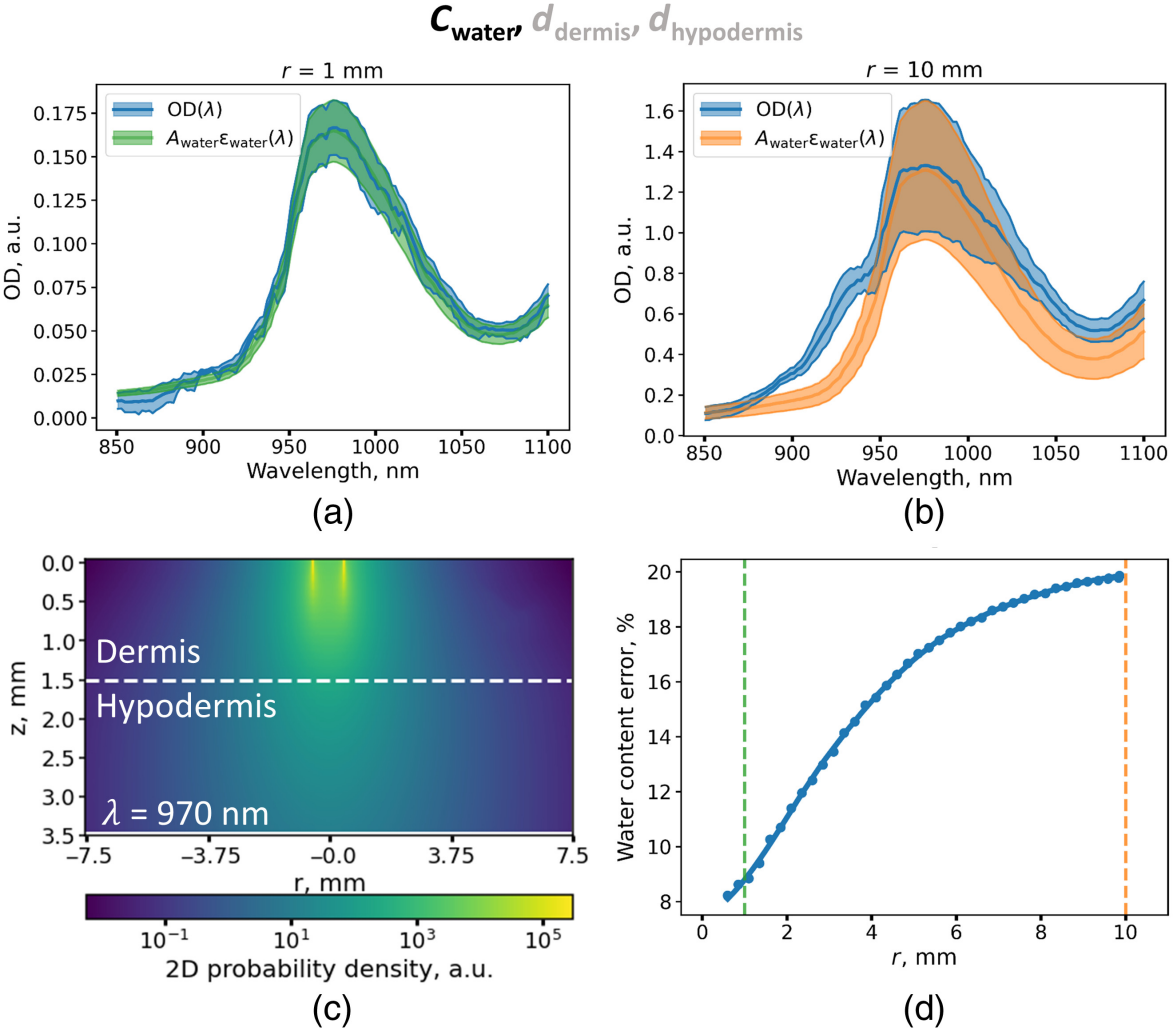
Determination of water content $c_{\text{water}}$ from simulated DRS for varying values of $d_{\text{dermis}}$ and $d_{\text{hypodermis}}$. Average (blue line) and standard deviation (blue region) of the OD spectra sets for distance between the fibers of 1 mm (a) and 10 mm (b). The average spectrum and standard deviation of the water spectral contribution obtained from fitting of the spectra at distances between the fibers of 1 mm [panel (a), green] and 10 mm [panel (b), orange]. (c) Density probability distribution for photon detection at a distance between the source and the detector of 1 mm for a model with dermal water concentration W=0.75 at a wavelength of 970 nm and with dermal thickness 1.5 mm. White dashed line corresponds to the border between dermis and hypodermis. (d) Average relative error of water concentration determined using the developed models as a function of the source–detector distance r. Each model uses data obtained at a single distance between the fibers.

The average simulated spectrum (denoted as OD) and the amplitude of the water-related component (denoted as $A_{\text{water}}$) and their standard deviation calculated for the set of models with a fixed water volume fraction of W=0.75 and varying thickness of the dermis and hypodermis for source-detector distances of 1 and 10 mm are shown in Figs. 4(a) and 4(b), respectively. As shown, the standard deviation of the water absorption band is significantly higher for large source–detector separation.

Next, the water content error was estimated at a distance of 1 mm between the fibers. The relative error of 8.6% in determining the water content was at a distance between the fibers of 1 mm and increased with the source-detector distance. It can be assumed that $A_{\text{water}}$ value is determined by both the water content in the dermis and dermis thickness at large distances between the fibers.

This suggestion is in agreement with the photon detection probability distribution [Fig. 4(c)], which allows estimating the fraction of detected photons from different depths. For typical system parameters (W=0.7, $d_{\text{dermis}}=1.5\;\mathrm{mm}$), calculations show that at a 1-mm source–detector separation, more than 80% of the signal is detected from the upper 1.1 mm of the skin. Since 80% of the response is formed in this layer, deeper layers do not make a significant contribution. Thus, with an increase in the dermal thickness above 1.1 mm, the response would not change significantly. On the contrary, with a change in water concentration in the dermis layer, one can expect a proportional increase in the peak of water absorption and amplitude $A_{\text{water}}$. Hence, it can be concluded that the amplitude $A_{\text{water}}$ at small distances is mainly determined by the water content in the skin (see Figure S3 in the Supplementary Material).

**Fig. 5:**
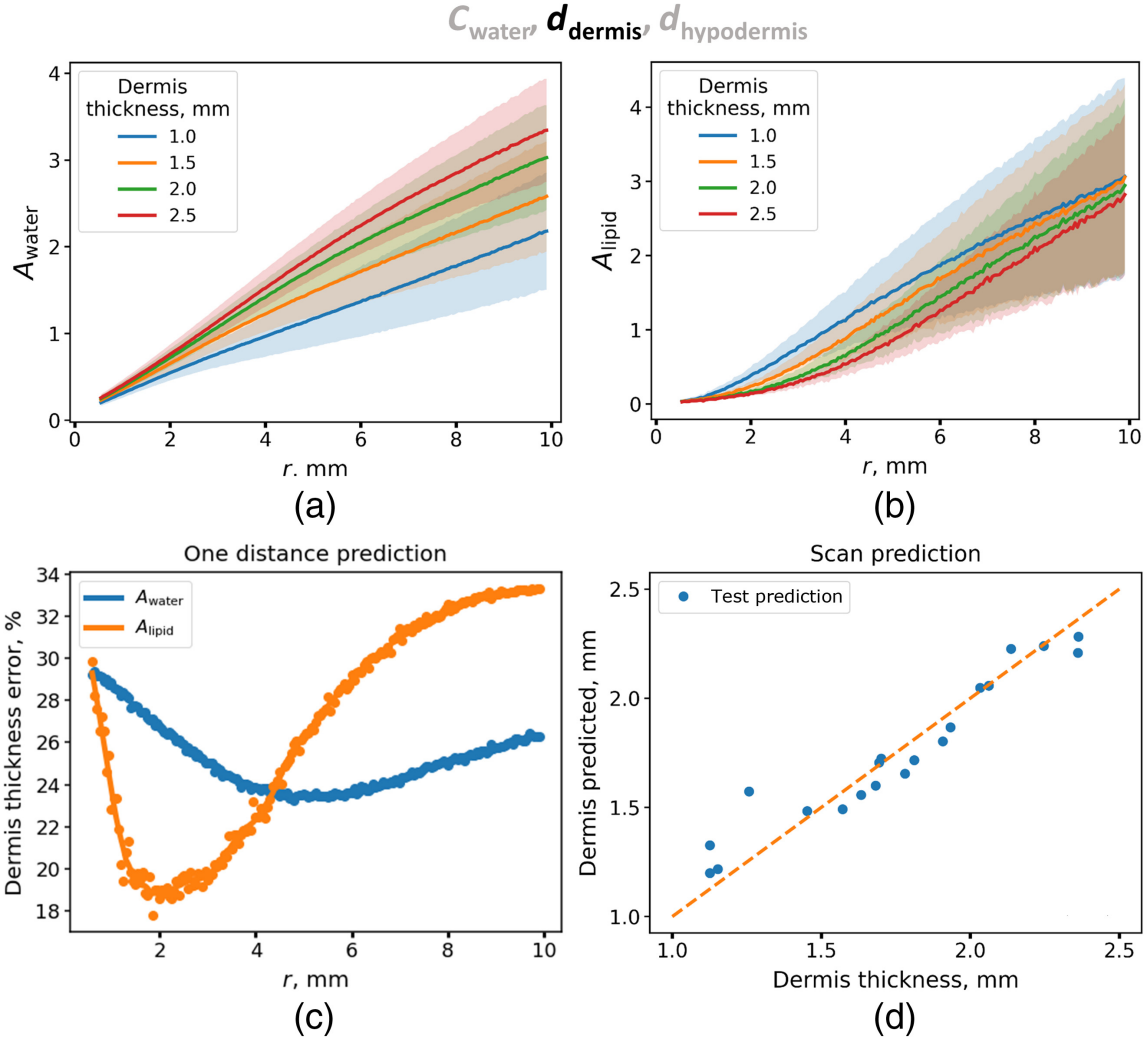
(a) Dependences of the water absorption amplitude $A_{\text{water}}$ on the distance between the source and the detector, obtained from the approximation of the calculated diffuse reflectance spectra for dermal thicknesses from 1 to 2.5 mm. (b) Dependences of the lipid absorption amplitude $A_{\text{lipid}}$ on the distance between source and detector fibers, obtained from the approximation of the calculated diffuse reflectance spectra for dermal thicknesses from 1 to 2.5 mm. (c) Dependence of the error in the prediction of the thickness of the dermis from the absorption amplitude of water $A_{\text{water}}$ (blue curve) and lipids $A_{\text{lipid}}$ (orange curve) obtained at different distances between the source and the detector from the corresponding distance. (d) Scatter plot for true and predicted values of dermal thickness obtained on the test set using a linear regression model with L2 regularization.

The second parameter of interest was the thickness of the dermis, i.e., of the first layer in the MCML simulation. First, the error of determining the dermal thickness from the DRS spectra at a single distance between the source and the detector was assessed. As can be seen in Fig. 5(a), distance dependences of $A_{\text{water}}$ for various dermal thicknesses differ significantly at large source-detector separation. The error in determining the thickness of the dermis $\delta_{\text{dermis}}(A_{\text{water}};r)$ as a function of distance was calculated for this class of models and is represented in Fig. 5(c). As can be seen, the smallest error is achieved at 5 mm and is ∼24%.

The smallest error of the dermal thickness determination using the $A_{\text{lipid}}$ value $\delta_{\text{dermis}}(A_{\text{lipid}};r)$ also demonstrated an error of about 19%. It was achieved for the interfiber distance of 2 mm [Fig. 5(c)]. To improve the accuracy of measuring the dermal thickness, a linear regression with the $L_{2}$ regularization that takes into account distance dependences of both $A_{\text{water}}$ and $A_{\text{lipid}}$ amplitudes was constructed, which yielded a 5% error of the dermal thickness determination [Fig. 5(d)].

**Fig. 6:**
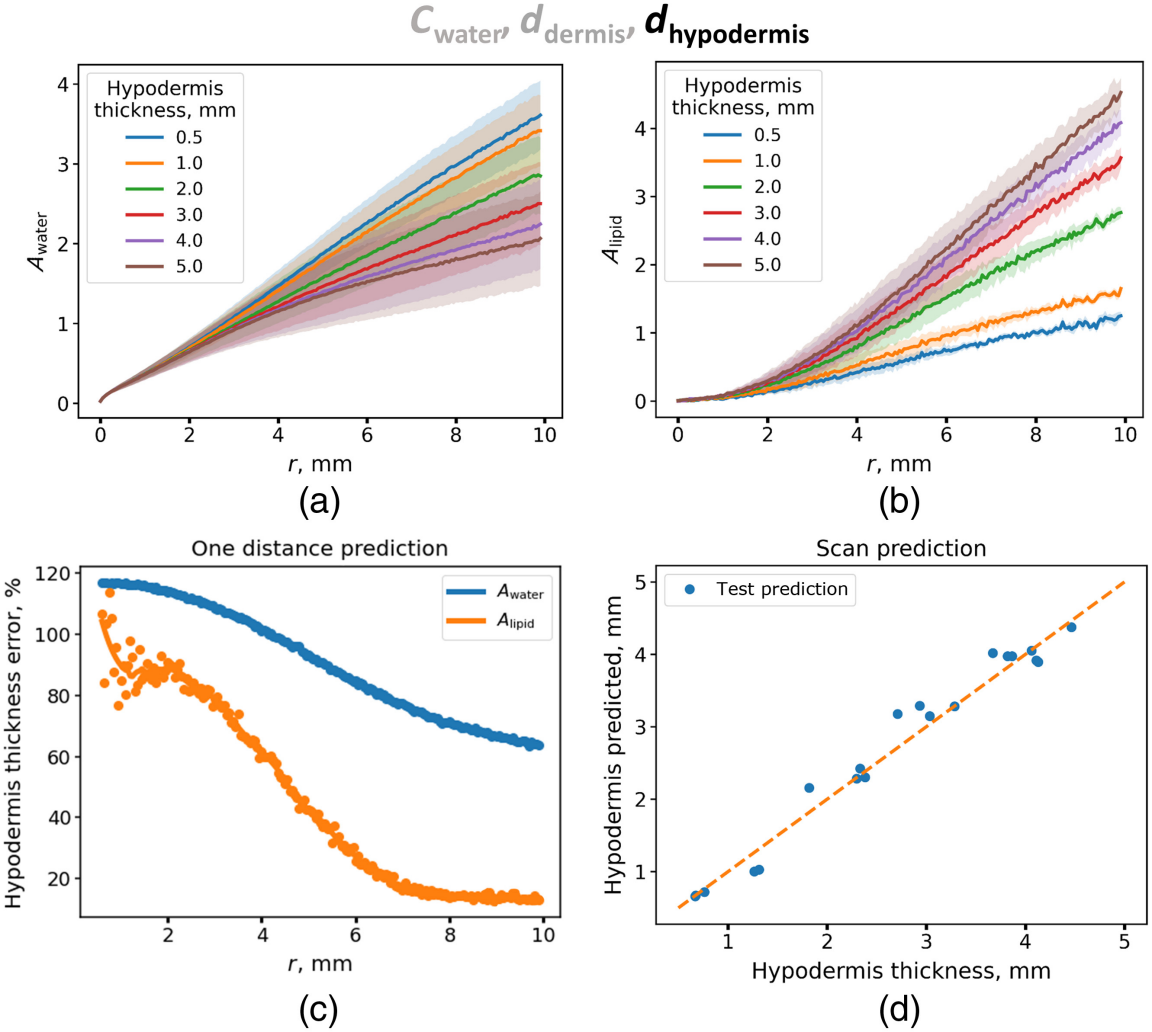
(a) Water absorption amplitude $A_{\text{water}}$ and (b) lipid absorption amplitude $A_{\text{lipid}}$ as a function of the distance between the source and the detector for different hypodermal thicknesses. (c) Dependence of the error in the prediction of the thickness of the hypodermis from the absorption amplitude of water $A_{\text{water}}$ (blue curve) and lipids $A_{\text{lipid}}$ (orange curve) obtained at different distances between the source and the detector from the corresponding distance. (d) Scatter plot for true and predicted values of hypodermal thicknesses obtained on the test set using a linear regression model with L2 regularization.

Similar procedure was carried out for determination of the hypodermal thickness. The error was estimated for the models that take into account data for a single distance between the source and the detector. As can be seen in Fig. 6(a), distance dependences of $A_{\text{water}}$ for various hypodermal thicknesses do not differ significantly. Therefore, using $A_{\text{water}}$ to determine the thickness of the hypodermis results in a high measurement error (>80%) [Fig. 6(c)]. Substantial difference in distance dependences of $A_{\text{lipid}}$ at large distances leads to better results. The error $\delta_{\text{hypodermis}}$ ($A_{\text{lipid}}$; r) as a function of distance has a minimum of 13.4%, which is achieved for the interfiber distance of 10 mm [Fig. 6(c)]. Linear regression with the $L_{2}$ regularization that takes into account the whole set of distance dependences of the $A_{\text{water}}$ and $A_{\text{lipid}}$ amplitudes was built to predict hypodermal thickness. In Fig. 6(d), the dependence between the hypodermal thickness from the test set and the hypodermal thickness predicted using machine learning approach is presented. Machine learning approach allowed us to reduce the relative error of hypodermal thickness determination to 6.3%.

This simulation shows that the simultaneous use of parameters determined from SR DRS can significantly decrease the error of the dermal and hypodermal thickness determination. These considerations were further used for the analysis of the experimental data.

### 3.3 Ultrasound Examination of the Skin Structure During Edema: Correlations with the SR DRS

**Fig. 7:**
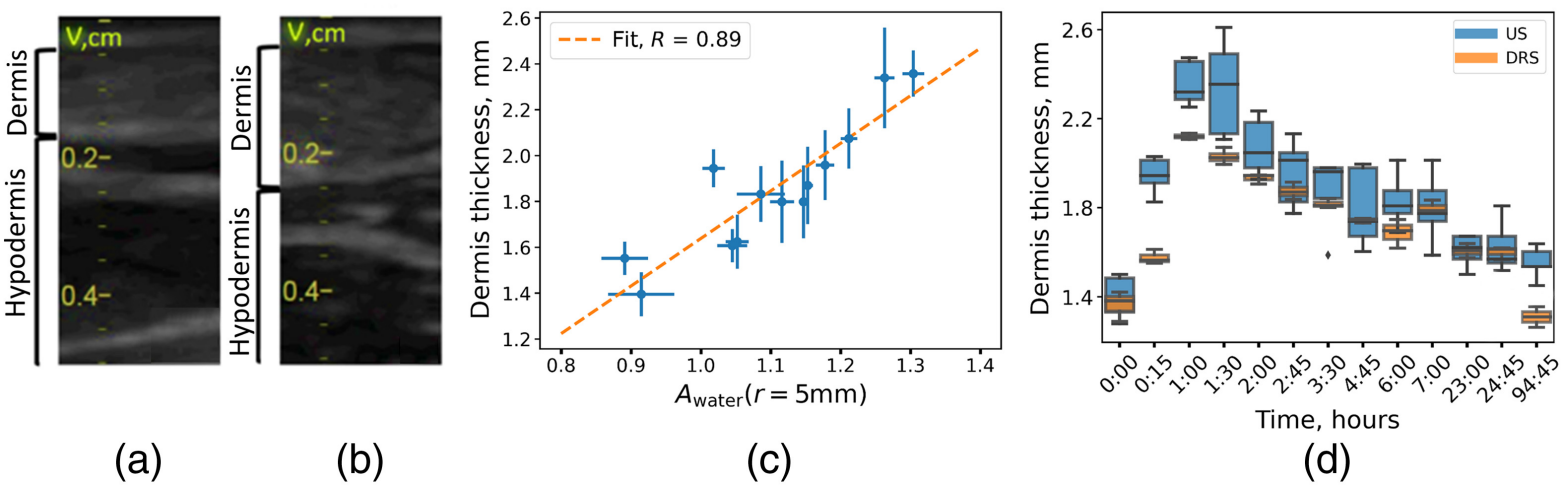
The US images of skin structure for normal (a) and edematous skin (b). (c) Dependence of the US-measured dermal thickness on the amplitude of water absorption $A_{\text{water}}$ obtained from the fitting of the diffuse reflectance spectra measured at a distance between the fibers of 10 mm. (d) Dermal thickness dynamics during edema determined by US and DRS (time axis is not to scale).

In addition to the SR DRS measurements presented in Fig. 3, a US study of cutaneous edema was performed to determine the thickness of the dermis. US images of the skin layers for the normal and edematous skin are shown in Figs. 7(a) and 7(b), respectively. Since the dermis and hypodermis have different acoustic properties, it is possible to discriminate between them in the US images.^33^

According to the US data, the thickness of the dermis increased from 1.4 to 2.2 mm during edema, that is consistent with the results from other studies.^34^ Since for the simulation data the minimum of $\delta_{\text{dermis}}(A_{\text{water}};r)$ was obtained at r=5  mm [Fig. 5(c)], this approach was applied to the experimental SR DRS data. It was found that the Pearson correlation coefficient between the dermal thickness measured by US and determined from the $A_{\text{water}}$ value was 0.89 [Fig. 7(c)]. The kinetics of the dermal thickness determined using the SR DRS and US upon histamine-induced edema are shown in Fig. 7(d).

It follows from the simulation data that the determination of water concentration from the $A_{\text{water}}$ amplitude has an error of 8% associated with the variability of other parameters [Fig. 4(d)]. Considering the fact that the thickness of the dermis increased from 1.4 to 2.2 mm during edema, it is likely that this variation will influence the $A_{\text{water}}$ value obtained at the minimum (1 mm) source–detector separation. From the data presented in Figs. 5(a) and S2, it can be deduced that such an increase is 15%. Thus, one can expect that out of a 33% increase in water contribution amplitude caused by edema [Fig. 3(e)], 15% is due to dermis swelling, and the rest is due to water content increase from 70% up to 81%. Hence, the correlation of the SR DRS parameters with dermal thickness has been demonstrated [Figs. 7(c) and 7(d)]. The observed dependence makes it possible to predict dermal thickness using experimental SR DRS data using measurements corresponding to higher values of interfiber distances. One of the limitations of the considered experiment is that it was performed for a single subject; therefore, we further evaluated the developed method by studying the skin parameters of healthy volunteers.

### 3.4 Skin Structure Assessment Using SR DRS: From Dermis to Hypodermis

US imaging and SR DRS measurements were performed for seven healthy volunteers. The measurements were carried out for different sites of the lower forearm: near the inner wrist, where the hypodermis layer is thin, near the antecubital fossa, where the hypodermis layer is thicker, and at the middle of the forearm. The thickness of the hypodermis, as determined from the US data, varied in the range of 0.5 to 4 mm for the tested subjects. The variability of the dermal thickness for volunteers was lower than for the case of edema. However, the thickness of the hypodermis layer observed in US images varied significantly.

**Fig. 8:**
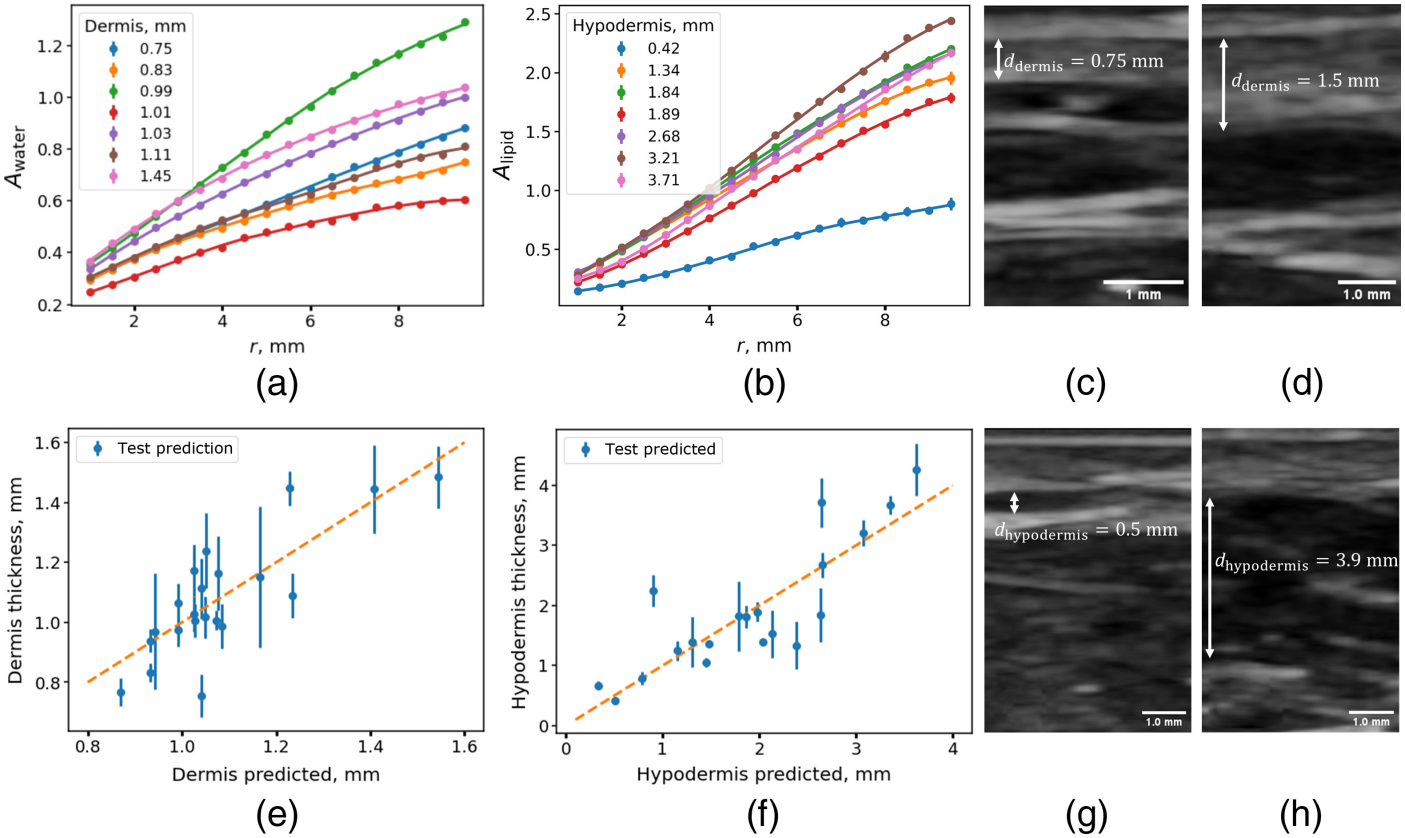
(a) Dependences of the water absorption amplitude $A_{\text{water}}$ on the distance between the source and the detector, obtained from the approximation of the diffuse reflectance spectra at different thicknesses of the dermis of volunteers. (b) Dependences of the lipid absorption amplitude $A_{\text{liid}}$ on the distance between the source and the detector, obtained from the fitting of diffuse reflectance spectra for various values of hypodermal thickness. (c,d) US images corresponding to minimal and maximal dermal thicknesses. (e) Dependence of US-measured dermal thicknesses on those predicted using a model trained on water and lipids dependencies on interfiber distance. (f) Dependence of US measured hypodermal thicknesses on those predicted from lipids contribution measured at a distance between the fibers of 10 mm. (g,h) US images corresponding to minimal and maximal hypodermal thicknesses.

To determine the thickness of the dermis from the SR DRS data, a model was built based on linear regression with L2 regularization. The $A_{\text{water}}$ and $A_{\text{lipid}}$ amplitudes obtained for all source-detector distances were used as the predictive features [Figs. 8(a) and 8(b)]. Dependence of US-measured dermal thickness on predicted values is presented in Fig. 8(e). The true value was considered the value obtained using US. Using the predicted values according to Eq. (5), the average relative error was calculated.

As a result, the average relative error of the dermal thickness prediction was 8.3%. Next, we focused on searching spectral descriptors indicative of the hypodermal thickness in our data. For this purpose, the $A_{\text{lipid}}$ amplitude at various distances between the source and detector fibers was estimated, and the dependence of the Pearson correlation coefficient between the thickness of the hypodermis and $A_{\text{lipid}}$ was calculated. Evidently, at distances between the fibers larger than 2 mm, there was a high correlation (>0.8) between the thickness of the hypodermis and $A_{\text{lipids}}$, reaching the maximum of 0.87 at r=10  mm (Figure S4 in the Supplementary Material). This result is in qualitative agreement with a result obtained for hypodermal thickness determination based on simulated data (Fig. 6). A linear regression was built to determine the thickness of the hypodermis from the experimental $A_{\text{lipids}}$ amplitude obtained at a distance of 10 mm. The resulting model allowed the determination of the thickness of the hypodermis with an average relative error of 23.4% and an average root mean squared error (RMSE) of 0.56 mm [Fig. 8(f)].

## 4 Conclusion

In the present work, using the SR DRS technique, the possibility of determining the physiological parameters of the skin was investigated *in vivo* for a model of cutaneous edema and for the normal skin. The parameters of interest included (1) the concentration of water in the dermis, (2) dermal thickness, and (3) the thickness of the hypodermis.

The propagation of light in the skin was simulated using the Monte Carlo method. From the simulation data, spatially resolved diffuse reflectance spectra were calculated using machine learning methods. Based on the simulation data, approaches for determining the water content in the dermis and thickness of the hypodermis and dermis were developed. It was shown that the minimum relative error of 9.3% in determining the water concentration is achieved at a distance between the source and the detector equal to 1 mm. The lowest error of hypodermal thickness determination was achieved at a distance of 10 mm and was 13.4%.

The developed procedure yielded a 0.89 Pearson correlation coefficient between the dermal thickness measured by US examination and amplitude of water absorption obtained using SR DRS during edema. Finally, machine learning models were built to determine the thickness of dermis and hypodermis of normal skin *in vivo* using SR DRS and US data with a relative error of 8.3% and 23.4%, respectively (Table 1). Thus, the developed method allows for a quantitative non-invasive relative assessment of molecular (water and lipids) and morphological (dermis and hypodermis thickness) parameters. Further development of this approach will be an important step in developing of a diagnostic method for assessing fluid retention in pathological conditions of heart failure and skin physiology for cosmetology and dermatology.

**Table 1:** Evaluation of models predicting physiological parameters of the skin using DRS data obtained in simulations and experimental measurements. The table represents RMSE and relative error of parameters, Pearson correlation coefficient R and p-value of non-correlation hypothesis for true and predicted values of parameters.

Parameters	RSME	Relative error (%)	R Pearson	p-value
Simulation results				
$C_{\text{water}}$	0.08 mm	9.3	0.89	$\ll 5\cdot 10^{-2}$
$d_{\text{dermis}}$	0.01 mm	5.0	0.97	$\ll 5\cdot 10^{-2}$
$d_{\text{hypodermis}}$	0.02 mm	6.3	0.99	$\ll 5\cdot 10^{-2}$
Experimental results				
$d_{\text{dermis}}$	0.11 mm	8.3	0.82	$5\cdot 10^{-6}$
$d_{\text{hypodermis}}$	0.56 mm	23.4	0.84	$3\cdot 10^{-6}$

## Code, Data, and Materials Availability

Data underlying the results presented in this paper are not publicly available at this time but may be obtained from the authors upon reasonable request.
