# Supplementary material for: Monitoring the skin structure during edema in vivo with spatially resolved diffuse reflectance spectroscopy
Source: J Biomed Opt. 2023 May 13;28(5):057002. doi: 10.1117/1.JBO.28.5.057002 (PMC10182858; doi:10.1117/1.JBO.28.5.057002)
Supplement: Supplementary file 1 [file JBO_028_057002_SD001.pdf]

## Supplemental Materials

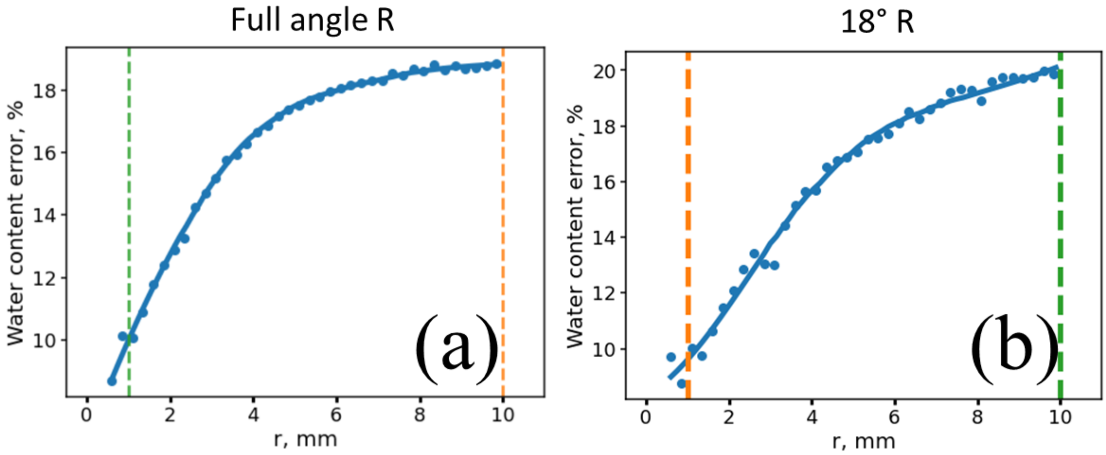


**Fig. S1.** Dependences of the average relative error in determining the water concentration $W_{pred}$ using the developed model on the source-detector separation r for with calculations of reflection coefficient R for two cases: (a) the reflectance is determined for photon packets, which exit the medium at a whole solid angle, (b) the reflectance is determined for photon packets that exit the medium at a solid angle of 18°.

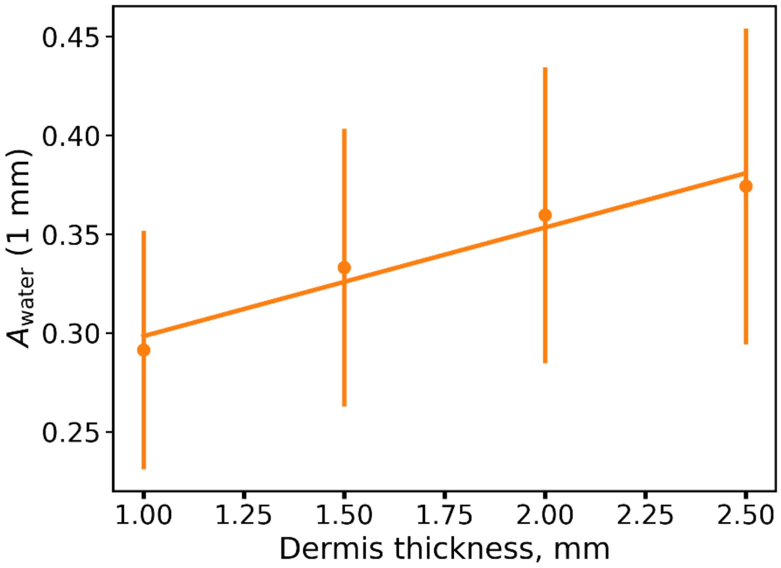


**Fig. S2.** Dependence of the amplitude of water $A_{water}$, obtained from the approximation of the calculated diffuse reflectance spectra for a distance between fibers of 1 mm, on the thickness of the dermis.

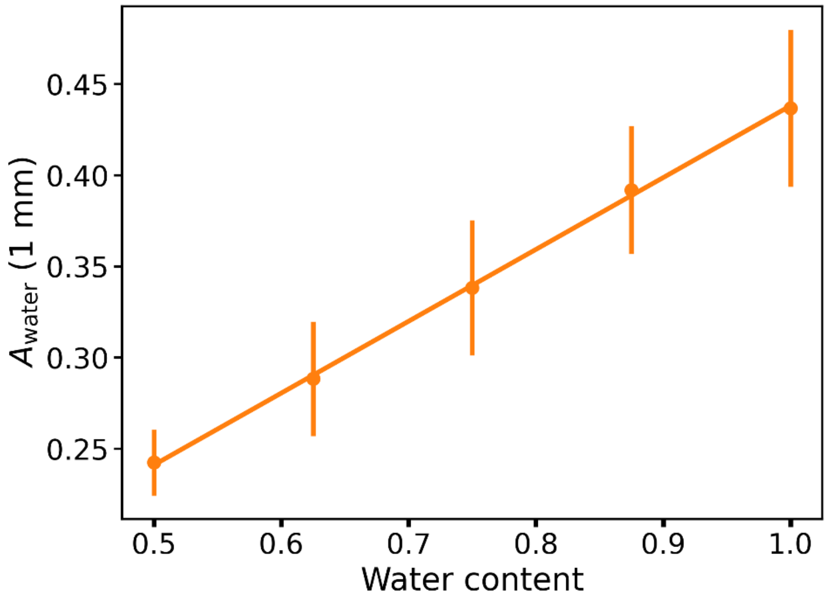


**Fig. S3.** Dependence of the amplitude of water $A_{water}$, obtained from the approximation of the calculated diffuse reflectance spectra for a distance between fibers of 1 mm, on the water fraction in first (dermal) layer W.

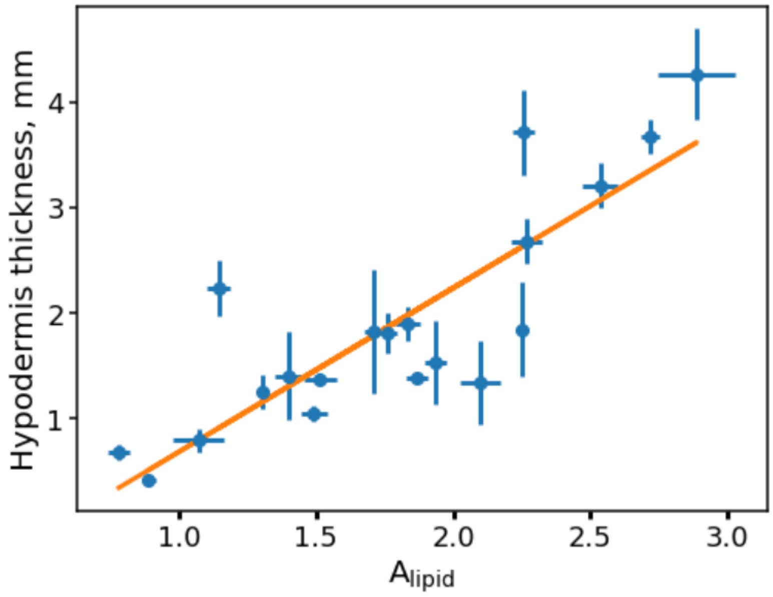


**Fig. S4.** Dependence of the hypodermal thickness, obtained from US measurements, on the amplitude of lipid $A_{lipid}$ obtained from the approximation of the measured diffuse reflectance spectra for a distance between fibers of 10 mm.
